# Description of Genetic Variants in *BRCA* Genes in Mexican Patients with Ovarian Cancer: A First Step towards Implementing Personalized Medicine

**DOI:** 10.3390/genes9070349

**Published:** 2018-07-11

**Authors:** Jesus Rolando Delgado-Balderas, Maria Lourdes Garza-Rodriguez, Gabriela Sofia Gomez-Macias, Alvaro Barboza-Quintana, Oralia Barboza-Quintana, Ricardo M. Cerda-Flores, Ivett Miranda-Maldonado, Hugo Mauricio Vazquez-Garcia, Lezmes Dionicio Valdez-Chapa, Mauro Antonio-Macedo, Michael Dean, Hugo A. Barrera-Saldaña

**Affiliations:** 1 Biochemistry and Molecular Medicine Department, School of Medicine, Universidad Autonoma de Nuevo Leon, Monterrey 64460, Mexico; jrolandodelgadob@gmail.com or jesus.delgadobl@uanl.edu.mx (J.R.D.-B.); lulugarza87@gmail.com or maria.garzarg@uanl.edu.mx (M.L.G.-R.); 2 Pathology Department, Hospital Universitario “Dr. Jose Eleuterio-Gonzalez”, Universidad Autonoma de Nuevo Leon, Monterrey 64460, Mexico; bpositivo66@hotmail.com (G.S.G.-M.); oralia.barbozaqnt@uanl.edu.mx (O.B.-Q.); imiranda77@hotmail.com (I.M.-M.); 3 Pathology Department, San Jose Tec Hospital, Monterrey 64718, Mexico; alvaro.barbosa@medicos.tecsalud.mx; 4 School of Nursing, Universidad Autonoma de Nuevo Leon, Monterrey 64460, Mexico; ricardocerda_mx@yahoo.com.mx; 5 Obstetrics and Gynecology Department, Christus Muguerza Hospital, Monterrey 64060, Mexico; drhugovazquezgarcia@gmail.com; 6 Gynecology and Obstetrics Department, Hospital Universitario “Dr. Jose Eleuterio Gonzalez”, Monterrey 64460, Mexico; lezmez@prodigy.net.mx (L.D.V.-C.); mauro8207@hotmail.com (M.A.-M.); 7 Laboratory of Translational Genomics, DCEG, National Cancer Institute, Bethesda, MD 20892, USA; deanm@mail.nih.gov; 8 Vitagenesis SA de CV, Monterrey 64630, Mexico

**Keywords:** *BRCA*, ovarian cancer, personalized therapy, sequencing

## Abstract

Gynecologic cancers are among the leading causes of death worldwide, ovarian cancer being the one with the highest mortality rate. Olaparib is a targeted therapy used in patients presenting mutations in *BRCA1* and *BRCA2* genes. The aim of this study was to describe *BRCA1* and *BRCA2* gene variants in Mexican patients with ovarian cancer. Sequencing of *BRCA1* and *BRCA2* genes from tumors of 50 Mexican patients with ovarian cancer was made in a retrospective, non-randomized, and exploratory study. We found genetic variants in 48 of 50 cases. A total of 76 polymorphic variants were found in *BRCA1*, of which 50 (66%) had not been previously reported. Furthermore, 104 polymorphic variants were found in *BRCA2*, of which 63 (60%) had not been reported previously. Of these polymorphisms, 5/76 (6.6%) and 4/104 (3.8%) were classified as pathogenic in *BRCA1* and *BRCA2*, respectively. We have described the genetic variants in *BRCA1* and *BRCA2* of tumors from Northeast Mexican patients with sporadic ovarian cancers. Our results showed that the use of genetic testing helps recognize patients that carry pathogenic variants which could be beneficial for personalized medicine treatments.

## 1. Introduction

Gynecologic cancers are among the leading causes of death worldwide, ovarian cancer being the one with the highest mortality rate, registering an incidence of 238,719 patients and 151,905 deaths in 2012, as reported by the World Health Organization (WHO) [1]. In the same year, Mexico had 3277 cases and 2105 people perished due to this disease. The trend for Mexico has been the same for the last 40 years. Compared with developing countries, Mexico has a low rate of cases [2]. Unfortunately, WHO has not updated the data on incidence and mortality rates for ovarian cancer in its electronic website. Recently, the estimated new cases and deaths of ovarian cancer for the United States of America for 2018: 22,240 and 14,070, respectively, were published [3].

Mortality is attributed to the absence of a sensitive and specific test that allows diagnosis in early stages [4,5]. To date, the American Cancer Society recommends screening with pelvic exploration, transvaginal ultrasound (TVUS), and detection of CA-125 for diagnosis of ovarian cancer, but this is possible only with biopsy of ovarian tissues [6]. Extensive studies have been conducted to determine the clinical utility of CA-125 and TVUS, however, results indicate a poor utility in reducing the incidence and mortality of ovarian cancer [7,8]*.* CA-125 is not a specific biomarker for ovarian cancer, it can be increased in other types of cancer and other specific non-malignant women’s conditions, such as pregnancy, pelvic inflammatory disease, and infectious disease [9,10,11]. High levels of CA-125 are detected in up to 80% of ovarian cancer patients with advanced disease and up to 60% in early stages. Moreover, its detection for clinical outcome is not recommended [5,12].

Beyond a specific diagnostic test, the lack of success for chemotherapy strategies plays a key role in ovarian cancer mortality. Recently, the Food and Drug Administration (FDA) approved a targeted therapy with the use of olaparib (Lynparza, AstraZeneca) that shows benefits in patients who carry mutations in any genes of homologous recombination, but mainly in *BRCA1* and *BRCA2* [13,14]. Olaparib inhibits the single strand DNA damage repair pathway through poly-ADP ribose polymerase (PARP) [15,16,17]. The use of this drug has demonstrated better objective response rates in platinum sensitivity patients with *BRCA1* and *BRCA2* mutations in phase II clinical trials and retrospective multicenter studies [18,19].

The refinement and cost reduction in Next Generation Sequencing technologies have allowed the expansion of genetic knowledge of human diseases [20,21]. Next Generation Sequencing applications have revealed the genomic landscape of the ovarian tumors types [22]. In this way, the mutations in the *BRCA* genes and their dysfunctions have helped drive clinical trials for olaparib administration in ovarian cancer patients [23]. 

Genomic variation findings of tumor driver genes are making it possible to personalize the treatment of cancer patients. Personalized cancer treatment includes early diagnosis, targeted therapy, monitoring response to treatment, tracking resistance, and prediction of disease-free survival. More recently, it has benefited from the isolation and analyses of circulating tumor DNA and Circulating Tumor Cells from plasma (liquid biopsies) [24,25,26]. 

The aim of this study was to describe genetic variants in the *BRCA1* and *BRCA2* genes in tumors from Northeast Mexican patients with sporadic ovarian cancer and to detect the percentage of potential eligible patients for future olaparib clinical trials.

## 2. Materials and Methods

### 2.1. Study Population and DNA Extraction

We conducted a retrospective, non-randomized, and exploratory study to search genetic variants in *BRCA* genes. The research protocol was approved by the Ethics Committee in Research of the Hospital Universitario at the Universidad Autonoma de Nuevo Leon (HU-UANL), with the registration number Bl13-005.

We collected cases from the Pathology Departments of public and private hospitals that participated in the study. Pathologists selected tumors from paraffin embedded tissues blocks for the identification of tumor cellularity of >80%. We ensured quality by avoiding areas of inflammation and necrosis. The patients have sporadic ovarian cancer. 

Tumor samples were obtained from 10 mg tissues and DNA was extracted using the AllPrep DNA/RNA FFPE kit (Qiagen, Hilden, Germany). DNA concentrations and quality (ratio 260 nm/280 nm, value ≥ 1.8) were evaluated by the NanoDrop 8000 Spectrophotometer (Thermo Scientific, Waltham, MA, USA).

### 2.2. DNA Sequencing

Complete sequencing of the exons of *BRCA1* and *BRCA2* genes was performed in the Ion Torrent Personal Genome Machine (PGM) Sequencer (Thermo Fisher Scientific, Waltham, MA, USA). Sequence reads were mapped to the hg19 reference genome and used to generate BAM files; variants were predicted using the both Torrent Suit Variant Caller (TSVC, Thermo Fisher Scientific, Waltham, MA, USA) and the Genome Analysis Toolkit (GATK, Intel Corporation, Santa Clara, CA, USA).

The sequencing process started with 30 ng of DNA, which was processed according to the standard protocol Multiplex Ion AmpliSeq *BRCA1* and *BRCA2* Panel (Life Technologies, Carlsbad, CA, USA). The panel amplifies 167 amplicons that cover about 16.3 kb and result in coverage between 98–100% of the coding regions of both genes. The libraries were designed according to the manufacturer by the platform Ion AmpliSeq Library Preparation Protocol (Life Technologies, Carlsbad, CA, USA). Each sample was labeled individually and added to the emulsion and subsequently sequenced using a P1 chip. Each run of that protocol produced about 10 Gb of information and each sample had an average depth of 500X [27]. 

### 2.3. Variant Calling, Filtering, and Annotation

Variants passing quality control and filtering were visually confirmed using the Integrative Genomics Viewer (IGV) [28]. Rare variants (with a reported frequency <1% or absent from the 1000 Genomes Project and from the Exome Aggregation Consortium (ExAC) populations) were examined as potential pathogenic mutations and annotated using Clininical Variation database (http://www.ncbi.nlm.nih.gov/clinvar). For missense variants, predictions of pathogenicity were generated using Align-GVGD (http://agvgd.iarc.fr/). 

## 3. Results

We included 50 tumors with a mean age of 51 years; the range was 33–86 years. The histological tumor subtypes were distributed as follows: 10 high-serous grade (20%), 13 endometrioid (26%), 7 papillary, 6 mucinous (13%), 1 low-serous grade (2%), 10 clear cell (20%), 1 transitional cell (2%), 1 dysgerminoma (2%), and 1 borderline (2%).

**Figure 1:**
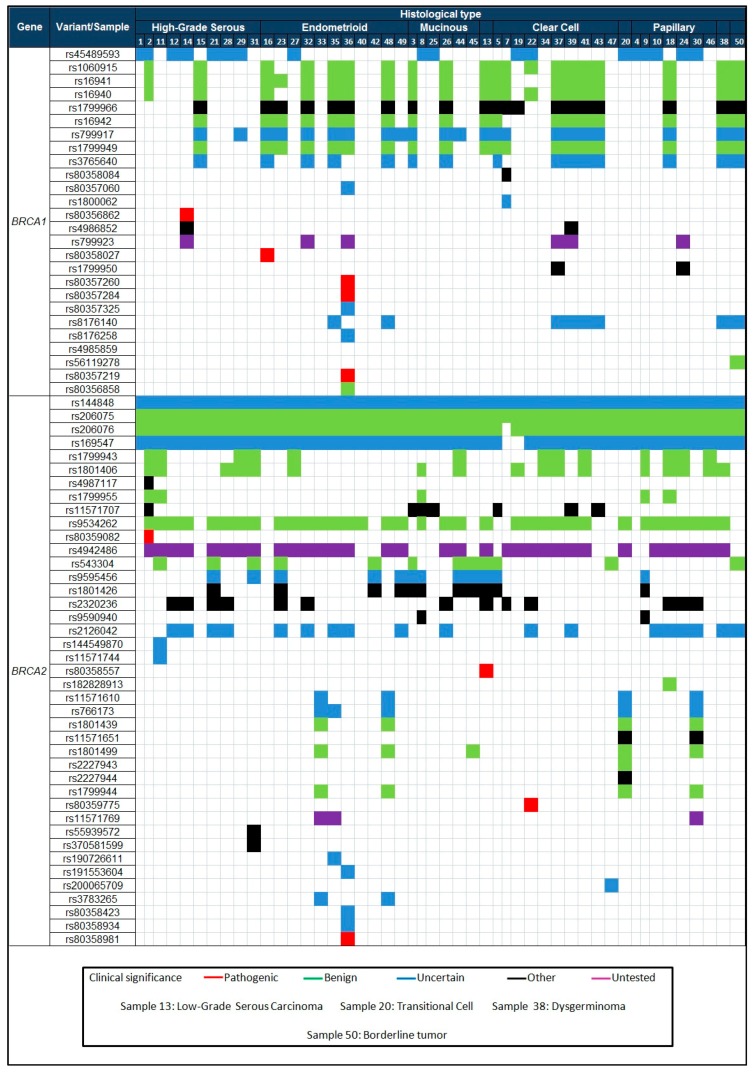
Variants reported for the *BRCA1* and *BRCA2* genes and their clinical significance by histological types.

We identified, in 48 of 50 (96%) cases, 76 polymorphic variants in *BRCA1* of which 26 (34%) had been previously reported. In *BRCA2*, we found 104 polymorphic variants of which 41 (39.4%) had also been reported previously, these variants are showed in Figure 1. The rest of the *BRCA1* and *BRCA2* variants found (66% and 60% respectively) had not been previously reported. Our samples have an average coverage of 650X.

We found pathogenic variants in *BRCA1* and *BRCA2*, 5/76 (6.6%) and 4/104 (3.8%), respectively. Table 1 shows pathogenic variants found in this study: 22% of variants were missense mutations, 55% nonsense, 11% intronic, 11% frameshift variants and 1% of deletions.

For samples with pathogenic variants, the histological subtype and age at diagnosis are shown in Table S1. The sample from patient 2 had a mixture of histological types: 90% high-grade serous carcinoma and 10% clear cell carcinoma; the sample from patient 13 was related to an early age of diagnosis (33 years old) with a histological type of low-grade serous carcinoma.

## 4. Discussion

In Mexico, the Federal Health Secretary’s prevention campaign directed for women focuses on breast and cervical cancers. Ovarian cancer is an urgent health problem for screening campaigns, taking advantage of new genomic technologies, such as Next Generation Sequencing methods, can help with better diagnoses and suggest new treatment approaches, such as personalized medicine [29,30,31].

The information reviewed in the Clinical Variation database showed that the *BRCA1* and *BRCA2* pathogenic variants found in our study have been reported in patients with breast cancer or ovarian cancer patients [32]. It is important to identify *BRCA1* and *BRCA2* pathogenic variants in ovarian cancer because they are therapeutic targets for olaparib therapy. 

We can see similarities in the *BRCA1* variants because rs1060915 (40%), rs16941 (40%), rs16940 (40%), rs1799966 (40%), rs16942 (36%), rs799917 (44%), rs1799949 (38%), rs3765640 (30%) are mostly present in samples with endometrioid and clear cell types. An unusual finding was the detection of 26 variants (four of them pathogenic) in endometrioid sample 36 (Figure 1 and Table S1). All histological types presented similarities in *BRCA2* with variants rs144848 (100%), rs206075 (100%), rs206076 (97.9%), rs169547 (95.8%), rs953426 (70.8%). Mutations in *BRCA* genes are rarely detected in the endometrioid histological type according to the COSMIC database [33]. The majority of mutations, variants, and epigenetic modifications in *BRCA1* are reported in the high-grade serous carcinoma samples [22]. 

The standard treatment for ovarian cancer is surgery and/or chemotherapy with platinum or taxanes, depending of the tumor phase; however, treatment with inhibitory PARPs can be improved if the genetic test reveals *BRCA* mutations. Patients can expect a better therapeutic response if they are platinum sensitive and are treated with combination therapy with inhibitory PARPs [34,35]. Patients who are carriers of *BRCA1* mutations have a better prognosis than those with epigenetic events [22].

Gelmon (2011) directed a phase 2, multicenter, open label, non-randomized study with 65 high-grade serous ovarian cancer patients: 17 were *BRCA* mutation carriers and only 7 patients (41%) had a positive response to treatment. On the other hand, 11 of 46 non-*BRCA* mutation carriers responded to olaparib[36]. Another study done by Ledermann (2012) included 265 patients (two groups: treatment and placebo). The treatment group with 136 patients had a complete objective response (criteria defined by Response Evaluation Criteria in Solid Tumors, RECIST) in 57 cases (41.9%). In this study, 25 cases (18.4%) were *BRCA1* mutation carriers and 6 cases had *BRCA2* mutations (4.4%) [37].

**Figure 2:**
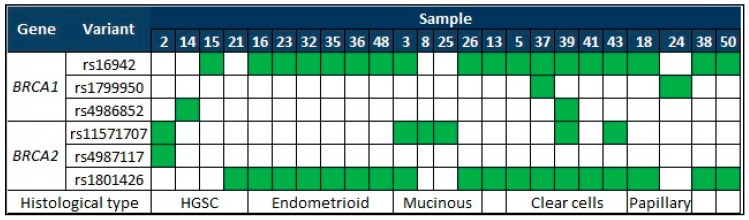
Genetic variants reported by Vaca-Paniagua [38] and our study. Samples: 13, Low-grade serous carcinoma; 38 Dysgerminoma; 50 Borderline tumor. HGSC: High-grade serous carcinoma.

Compared with a previous Mexican study of breast/ovarian hereditary cancer patients, we found six variants in common between the studies, all of them benign. These results are shown in Figure 2 [38].

**Figure 3:**
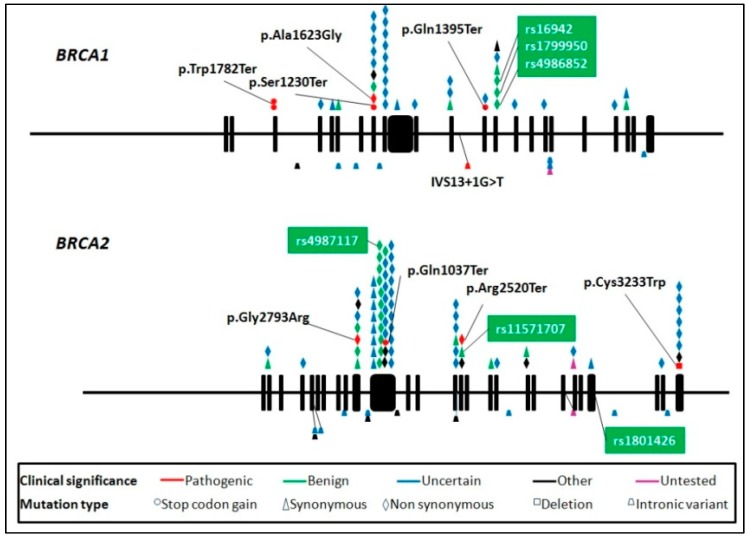
Mutations found in *BRCA* genes. Amino acid changes for the pathogenic variants are shown. Boxes correspond to the variants that coincide with those reported by Vaca-Paniagua [38].

Figure 3 represents the global distributions of variants found in our study across both genes. Variant type and clinical significance and variants previously reported in Mexican ovarian cancer patients are shown [38]. 

Villarreal-Garza (2014) reported the clinical significance of *BRCA* mutations in ovarian and breast cancer patients with hereditary cancer history. They found *BRCA* mutations in 26 of 98 ovarian cancer patients (28%), also reporting the Mexican founder mutation *BRCA1* ex9-12del in 35% of their patients from central Mexico [39]. In comparison, we found pathogenic variants on *BRCA* genes in 12% of selected patients. This percentage is important because it corresponds to patients with sporadic cancer and shows the importance of molecular screenings in all patients for better diagnosis and therapy strategies.

Another important aspect to highlight is the difference in the molecular test for screening the genetic variants in the *BRCA* genes. For example, we used Multiplex Ion AmpliSeq *BRCA1* and *BRCA2* Panel that includes primers for the entire coding region, whilst Villarreal-Garza and colleagues used HISPANEL for Sequenom MassARRAY (Sequenom Inc., San Diego, CA, USA) and end point PCR for *BRCA1* ex9-12del mutation. Nevertheless, the founder mutation *BRCA1* ex9-12del was absent in our study likely because we have samples from sporadic cancer patients from the Northeast of Mexico, in comparison with other studies that include patients with hereditary history of cancer and are from central Mexico (Mexico City and Puebla State), the region of origin of this founder mutation [39,40,41]. Additionally, our methodology is not optimal for searching ex9-12del of *BRCA1* because this is a large rearrangement and our samples are from FFPE tissues with degraded DNA.

Mutation screenings in druggable driver genes are of great value for medical treatment decisions in over 50% of ovarian cancer patients. For example, there are therapies for patients that carry mutations in *BRCA* (olaparib), *NF1* (temsirolimus), *PI3K/RAS* (sorafenib), or that exhibit HER2 over expression (trastuzumab) [42]. The next step in the implementation of personalized medicine of ovarian cancer is the search for these genetic alterations with Next Generation Sequencing, real time PCR, droplet digital PCR, or Sanger Sequencing through the use of liquid biopsies from blood, urine, or liquid Pap smears.

The Mexican population is genetically diverse, consisting of peoples of Amerindian, European, African and Asian descent and detailed ancestry studies have been described by other Mexican research groups [43]. We have reported the diversity of polymorphism frequencies for the *CYP2D6*, *CYP3A5*, *CYP2C8*, and *IL-10* genes in breast cancer patients from Mexico and Spain and classified them by metabolic activity for chemotherapy but no specific function has been described for these polimorphisms [44]. Previously, we identified polymorphisms associated with the metabolism of atorvastatin with the aim of finding pharmacokinetic biomarkers in the Mexican population [45]. 

Our study reports for the first time the genetic diversity of *BRCA1*and *BRCA2* genes in tumors from Northeast Mexican ovarian cancer patients. In addition, we report pathogenic variants in these genes according to the ClinVar database (accessed May, 2018).

This work raises the need to implement molecular genetic testing with high specificity, sensitivity, and low cost for all ovarian cancer patients. Test results may help oncologists offer more precise molecular diagnosis and better personalized therapies. In this case, 12% of patients could be candidates for olaparib therapy. 

Dean et al. showed the importance of performing screening of genetic variants along *BRCA* genes in Hispanic populations. These populations have limited economic resources to access genomic medicine for better prognosis and treatment of ovarian cancer [27].

Comprehensive studies that include high-quality samples from biobanks, complete clinical records, and better bioinformatics analyses are very important to identify key factors for the diagnosis, treatment, and follow-up of the therapeutic response of ovarian cancer patients [46,47]. 

## 5. Conclusions

We have described genetic variants in *BRCA1* and *BRCA2* from tumors of Northeast Mexican patients with sporadic ovarian cancer. Our results showed that the use of genetic testing helps identify patients that carry pathogenic variants that could beneficial for personalized medicine treatment. 

## Figures and Tables

**Table 1:** Pathogenic variants in *BRCA1* and *BRCA2* in Mexican ovarian cancer patients.

Gene	Variants	HGVS	Amino Acid Change	Genomic Location (GHCh38)	Type
*BRCA1*	rs80356862	4868C>G	A1623G	Chr17:43071046	Missense variant
	rs80358027	c.4357+1G>A	IVS13+1G>A	Chr17:43082403	Splice donor variant
	rs80357260	c.4183C>T	Q1395*	Chr17:43090946	Nonsense variant
	rs80357284	c.5346G>A	W1782*	Chr17:43049181	Nonsense variant
	rs80357219	c.5345G>A	W1782*	Chr17:43049182	Nonsense variant
*BRCA2*	rs80359082	c.8377G>A	G2793R	Chr13:32370447	Missense variant
	rs80358557	c.3109C>T	Q1037*	Chr13:32337464	Nonsense variant
	rs80359775	c.9699_9702delTATG	C3233Wfs	Chr13:32398212 - 32398215	Frameshift variant
	rs80358981	c.7558C>T	R2520*	Chr13:32356550	Nonsense variant

*: Codon stop gain.

## Supplementary Materials

The following are available online at http://www.mdpi.com/2073-4425/9/7/349/s1. Table S1: Samples with pathogenic variants in both genes.
