# Supplementary material for: Description of Genetic Variants in BRCA Genes in Mexican Patients with Ovarian Cancer: A First Step towards Implementing Personalized Medicine
Source: Genes (Basel). 2018 Jul 11;9(7):349. doi: 10.3390/genes9070349 (PMC6071230; doi:10.3390/genes9070349)
Supplement: Supplementary file 1 [file genes-09-00349-s001.pdf]

Table S1. Pathogenic variants found in both *BRCA* genes per sample and its histological tumor type.

Gene	SNP	Patient number/histological tumor type					
		2/High-grade serous	14/ High-grade serous	16/Endometrioid	36/ Endometrioid	13/Low-grade serous	22/Clear cell
BRCA1	rs80356862		59 years				
	rs80358027			41 years			
	rs80357260				68 years		
	rs80357284						
	rs80357219						
BRCA2	rs80359082	55 years					
	rs80358557					33 years	
	rs80359775						43 years
	rs80358981						
